# Survey of bacterial diversity in chronic wounds using Pyrosequencing, DGGE, and full ribosome shotgun sequencing

**DOI:** 10.1186/1471-2180-8-43

**Published:** 2008-03-06

**Authors:** Scot E Dowd, Yan Sun, Patrick R Secor, Daniel D Rhoads, Benjamin M Wolcott, Garth A James, Randall D Wolcott

**Affiliations:** 1 United States Department of Agriculture ARS Livestock Issues Research Unit, Lubbock, TX, USA; 2 Medical Biofilm Research Institute, Lubbock, TX, USA; 3 Center for Biofilm Engineering, Montana State University, Bozeman, MT, USA

## Abstract

**Background:**

Chronic wound pathogenic biofilms are host-pathogen environments that colonize and exist as a cohabitation of many bacterial species. These bacterial populations cooperate to promote their own survival and the chronic nature of the infection. Few studies have performed extensive surveys of the bacterial populations that occur within different types of chronic wound biofilms. The use of 3 separate16S-based molecular amplifications followed by pyrosequencing, shotgun Sanger sequencing, and denaturing gradient gel electrophoresis were utilized to survey the major populations of bacteria that occur in the pathogenic biofilms of three types of chronic wound types: diabetic foot ulcers (D), venous leg ulcers (V), and pressure ulcers (P).

**Results:**

There are specific major populations of bacteria that were evident in the biofilms of all chronic wound types, including *Staphylococcus, Pseudomonas, Peptoniphilus, Enterobacter, Stenotrophomonas, Finegoldia*, and *Serratia *spp. Each of the wound types reveals marked differences in bacterial populations, such as pressure ulcers in which 62% of the populations were identified as obligate anaerobes. There were also populations of bacteria that were identified but not recognized as wound pathogens, such as *Abiotrophia para-adiacens *and *Rhodopseudomonas *spp. Results of molecular analyses were also compared to those obtained using traditional culture-based diagnostics. Only in one wound type did culture methods correctly identify the primary bacterial population indicating the need for improved diagnostic methods.

**Conclusion:**

If clinicians can gain a better understanding of the wound's microbiota, it will give them a greater understanding of the wound's ecology and will allow them to better manage healing of the wound improving the prognosis of patients. This research highlights the necessity to begin evaluating, studying, and treating chronic wound pathogenic biofilms as multi-species entities in order to improve the outcomes of patients. This survey will also foster the pioneering and development of new molecular diagnostic tools, which can be used to identify the community compositions of chronic wound pathogenic biofilms and other medical biofilm infections.

## Background

Biofilms are well documented as medical problems associated with implants [1-9] and certain diseases [10-17]. However, the nature and importance of chronic wound pathogenic biofilms (CWPB) is only now beginning to be realized as reviewed and discussed in the scientific literature [18-25]. Chronic wounds, including diabetic foot ulcers (D), venous leg ulcers (V), and pressure ulcers (P), are often resistant to natural healing and require long term medical care [26-38]. Chronic wounds and their associated pathogenic biofilms [20,22,24,39-44] are also associated as a primary contributing factor in hundreds of thousands of annual deaths and billions of dollars in direct medical costs annually [20,22,38,45-55].

For decades, medical microbiologists have relied on culture techniques to elucidate the complexity of infections including CWPB [56,57]. These techniques with only minor advancements have been used over the past 150 years and are currently the mainstay of the clinical microbiology laboratories. These culture methods can be used to identify the "culturable" bacteria associated with such biofilms. However, the use of laboratory culture techniques is typically only able to detect (as isolates) those organisms which grow relatively quickly and easily in laboratory media. This presents an important problem and descrepency because many of the bacteria in wound biofilms are recalcitrant to culture [58]. Thus, there is a lack of information about the diversity of populations that occur in association with CWPB. A better understanding of bacterial populations associated with CWPB is necessary to enable development of next generation management and therapeutics [59-62].

No studies have been identified which have utilized deep sequencing molecular methods (pyrosequencing) to evaluate the diversity of microbial populations that occur within the pathogenic biofilms associated with each of the three major types of chronic wounds. This report describes the first use of partial ribosomal amplification and pyrosequencing (PRAPS) to look at the microbial diversity in chronic wounds. Combined with two more traditional molecular methods; full ribosomal amplification, cloning and Sanger sequencing (FRACS) and partial ribosomal amplification, density gradient gel electrophoresis (DGGE) and Sanger sequencing (PRADS) we are providing a comprehensive survey of the microbial populations that are present in three types of chronic wound biofilms: venous leg ulcers, diabetic foot ulcers, and pressure ulcers. The compilation of data obtained with each of these methods provides one of the first comprehensive surveys of bacteria that are occur in three different types of chronic wound biofilms.

## Results and Discussion

### Comparison of Molecular Methods and wound types

**Figure 1:**
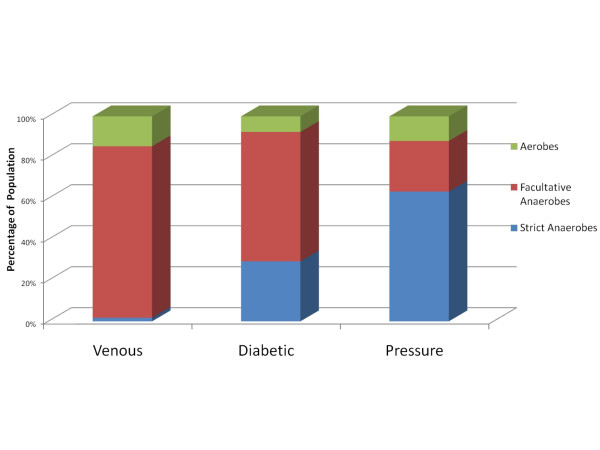
**Distribution of Bacterial Populations in Chronic Wounds in Relation to Aerotolerance**. Diabetic, venous, or pressure ulcer types were analyzed separately using pyrosequencing and the resulting populations grouped into 3 catagories based upon their suggested aerotolerance. This figure graphically illustrates the relative distribution of these functional catagories among the wound types.

It should be noted that this paper is not intended to contrast each of the molecular methods, or to purposefully compare wound types, but rather to detail the results of each individually in the hopes of gaining an understanding of the microbial diversity within pathogenic biofilms. Although this study could be used to compare or contrast the three molecular methods, we sought instead to use these relatively different strategies to better survey and report the diversity in the different types of wounds. A portion of the bias of one molecular method (e.g. due to primer specificity and universality) we intended to be somewhat compensated by the other methods, each of which utilize different "universal" 16S primers. Another important note is that these analyses do not represent the diversity within a given wound from a single patient; instead these data represented diversity among a given chronic wound type. In-depth comparison of the populations between each wound type as part of this study would also have been outside the scope of the methodologies and experimental design employed. The primary observations that could be logically employed when comparing wound types are two-fold. Each wound type demonstrated populations and diversity that were markedly more prevalent than those seen in other wound types as discussed below. Each wound group also demonstrated a different level of oxygen tolerance among its bacterial populations (Figure 1). This second observation indicates there may be a common pathophysiology among wound types that likely affects the ecology of the wound environment and may play an important role in determining the bacterial genera that can become integrated as part of a wound biofilm. These observations obviously cannot be fully addressed within the scope of this survey yet they do provide important directions for future research.

### PRAPS

The Rowher lab [63] was the first to pyrosequencing approaches to evaluate the diversity of complex microbial ecosystems by looking at environmentally isolated genome sequences from two sites in the Soudan Mine, Minnesota, USA. A few studies have utilized a PRAPS approach to evaluate the genetic diversity of clinical samples [64] or as as a form of clinical isolate genotyping [65-69]. To date, PRAPS has not been used to evaluate the biodiversity of clinically infected biofilm samples, particularly CWPB.

A total of 193890 sequences were generated among the 4 samples including the pooled control sample of which over 129,000 sequences were utilized in the actual PRAPS analysis, gave a comprehensive functional survey of pathogenic biofilm populations within each wound type (Table 1). Facultative gram negative rods predominated in the V wounds. The predominant bacterial types in V biofilms were *Enterobacter, Serratia, Stenotrophomonas*, and *Proteus *spp. (Table 2). Strict anaerobes, cocci, and gram positives were scarce. In D samples the primary bacterial genera were *Staphylococcus, Peptoniphilus, Rhodopseudomonas, Enterococcus, Veillonella, Clostridium*, and *Finegoldia *spp. Facultative and strict anaerobic gram positive cocci were most prevalent (Table 3). Finally, in P samples, the predominant species were *Peptoniphilus, Serratia, Peptococcus, Streptococcus*, and *Finegoldia *spp. Thus, strict anaerobic gram positive cocci dominated the communities within P biofilms (Table 4).

**Table 1:** Overview of the phenotypes of microbial populations as determined using pyrosequencing (PRAPS).

	**Pressure Ulcer seq**	**Pressure Ulcer genus**	**Diabetic Foot Ulcer seq**	**Diabetic Foot Ulcer genus**	**Venous Let Ulcer seq**	**Venous Leg Ulcer genus**
Anaerobe	17105	12	10519	15	523	6
Aerobe	3241	9	2740	12	4402	9
Facultative anaerobes	6686	15	22673	19	25226	16
Gram positive	18751	18	22821	20	984	12
Gram negative	8281	17	13111	26	29167	19
Rod	8384	25	14682	34	29729	25
Cocci	18648	12	21250	12	422	6
UWB	440	8	656	7	1724	3

The data from pyrosequencing (PRAPS) is broken down into the number of sequences (seq) from each sample associated with the indicated phenotypic characteristic (anaerobe, gram positive, etc). Also shown are the number of genera that were associated with each of the phenotypic categories. UWB refers to unknown wound bacteria or sequences without significant similarity to a database sequences.

**Table 2:** Results obtained for venous leg ulcer sample using pyrosequencing (PRAPS). This table provides the identified genera of the bacteria found using pyrosequencing (PRAPS) in the V sample, the number of sequences corresponding to a given genus, the known gram staining properties, the aerotolerance (anaerobic, anaerobic, or facultative anaerobic) nature of the genus, and the relative shape (rod or cocci) of the genus.

**Genus**	**Seq**	**%**	**Gram**	**Aerotolerance**	**Shape**
*Enterobacter *spp.	14288	44.83	-	Facultative anaerobe	Rod
*Serratia *spp. *	6132	19.24	-	Facultative anaerobe	Rod
*Stenotrophomonas *spp.	3532	11.08	-	Aerobe	Rod
*Proteus *spp.	2469	7.75	-	Facultative anaerobe	Rod
UWB	1446	4.54	unk	Unk	Unk
*Proteus mirabilis.*	1080	3.39	-	Facultative anaerobe	Rod
*Salmonella *spp.	739	2.32	-	Facultative anaerobe	Rod
*Clostridium *spp.	408	1.28	+	Anaerobe	Rod
*Alcaligenes faecalis*	337	1.06	-	Aerobe	Rod
UWB	261	0.82	unk	Unk	Unk
*Pseudomonas *spp.*	185	0.58	-	Aerobe	Rod
*Staphylococcus *spp.*	143	0.45	+	Facultative anaerobe	Cocci
*Brevundimonas diminuta*	123	0.39	-	Aerobe	Rod
*Streptococcus *spp.	107	0.34	+	Facultative anaerobe	Cocci
*Acinetobacter *spp.	102	0.32	-	Aerobe	Rod
*Enterococcus *spp.	94	0.29	+	Facultative anaerobe	Cocci
*Pantoea *spp.	81	0.25	-	Facultative anaerobe	Rod
Corynebacterium striatum*	81	0.25	+	Aerobe	Rod
*Peptoniphilus *spp.	65	0.20	+	Anaerobe	Cocci
*E. coli*	31	0.10	-	Facultative anaerobe	Rod
*Bacillus *spp.	25	0.08	+	Aerobe	Rod
*Paenibacillus *spp.	24	0.08	+	Facultative anaerobe	Rod
*Eubacterium *spp.	24	0.08	+	Anaerobe	Rod
*Klebsiella *spp.	21	0.07	-	Facultative anaerobe	Rod
*Xanthomonas *spp.	17	0.05	-	Aerobe	Rod
UWB	17	0.05	unk	Unk	Unk
*Ferrimonas *spp.	17	0.05	-	Facultative anaerobe	Rod
*Finegoldia magna*	13	0.04	+	Anaerobe	Cocci
*Dendrosporobacter quercicolus*	13	0.04	-	Anaerobe	Rod

UWB refers to unknown wound bacteria or sequences without significant similarity to database sequences. * The asterisk identifies bacteria previously cultured from a single culture result from each subject's medical record from subjects in the V pool. The only bacterium cultured that was not found using PRAPS was *Citrobacter*. The % represents the percentage of the total sequences analyzed within the sample.

**Table 3:** Results obtained for diabetic foot ulcer sample using pyrosequencing (PRAPS).

**Genus**	**Seq**	**%**	**Gram**	**Aerotolerance**	**Shape**
*Staphylococcus *spp.	10874	29.72	+	Facultative anaerobe	Cocci
*Peptoniphilus *spp.	2555	6.98	+	Anaerobic	Cocci
*RhodoPseudomonas *spp.	2541	6.94	-	Facultative anaerobe	Rod
*Enterococcus *spp.	2341	6.40	+	Facultative anaerobe	Cocci
*Veillonella *spp.	1978	5.41	-	Anaerobic	Cocci
*Clostridium *spp.	1975	5.40	+	Anaerobic	Rod
*Finegoldia magna*	1953	5.34	+	Anaerobic	Cocci
*Haemophilus *spp.	1701	4.65	-	Facultative anaerobe	Rod
*Acinetobacter *spp.	1301	3.56	-	Aerobic	Rod
*Morganella *spp.	1240	3.39	-	Facultative anaerobe	Rod
*Serratia *spp.	1125	3.07	-	Facultative anaerobe	Rod
*Proteus *spp.	1072	2.93	-	Facultative anaerobe	Rod
*Dialister *spp.	1029	2.81	-	Anaerobic	Rod
*Streptococcus *spp.	751	2.05	+	Facultative anaerobe	Cocci
*Stenotrophomonas *spp.	669	1.83	+	Aerobe	Rod
*Peptococcus niger*	588	1.61	+	Anaerobic	Cocci
UWB	342	0.93	unk	Unk	Unk
*Klebsiella *spp.	326	0.89	-	Facultative anaerobe	Rod
*Actinomyces *spp.	307	0.84	+	Facultative anaerobe	Rod
*Gordonia *spp.	302	0.83	+	Aerobic	Rod
*Delftia *spp.	251	0.69	-	Aerobic	Rod
*Gemella *spp.	168	0.46	+	Anaerobic	Cocci
*Corynebacterium *spp.	157	0.43	+	Facultative anaerobe	Rod
UWB	143	0.39	unk	Unk	Unk
UWB	107	0.29	unk	Unk	Unk
*Salmonella enterica*	102	0.28	-	Facultative anaerobe	Rod
*Fusobacterium *spp.	99	0.27	-	Anaerobic	Rod
*Varibaculum cambriense*	54	0.15	+	Anaerobic	Rod
*Enterobacter *spp.	52	0.14	-	Facultative anaerobe	Rod
*Bacillus *spp.	51	0.14	+	aerobic	Rod
*Eikenella *spp.	42	0.11	-	facultative anaerobe	Rod
*Anaerococcus *spp.	42	0.11	+	anaerobic	Cocci
*Hydrogenophaga *spp.	40	0.11	-	aerobic	Rod
*Alcaligenes faecalis*	36	0.10	-	aerobic	Rod
*E coli*	32	0.09	-	facultative anaerobe	Rod
*Sphingomonas *spp.	26	0.07	-	aerobic	Rod
*Acidovorax *spp.	26	0.07	-	aerobic	Rod
*Prevotella *spp.	22	0.06	-	anaerobic	Rod
UWB	20	0.05	unk	unk	Unk
*Eubacterium *spp.	20	0.05	+	anaerobic	Rod
*Bacteroides *spp.	20	0.05	-	anaerobic	Rod
UWB	17	0.05	unk	unk	Unk
UWB	16	0.04	unk	unk	Unk
*Selenomonadaceae *spp.	16	0.04	-	anaerobic	Rod
*Brevibacterium *spp.	14	0.04	+	aerobic	Rod
*Riemerella *spp.	13	0.04	-	aerobic	Rod
UWB	11	0.03	unk	unk	Unk
*Bradyrhizobium *spp.	11	0.03	-	aerobic	Rod
*Pantoea agglomerans*	10	0.03	-	facultative anaerobe	Rod

This table provides the identified genus of the bacteria found using pyrosequencing (PRAPS) in the D sample, the number of sequences corresponding to this genus, the known gram staining properties, the aerotolerance (anaerobic, anaerobic, or facultative anaerobic) nature of the genus, and the shape of the genus. UWB refers to unknown wound bacteria or sequences without significant similarity to database sequences. * The asterisk identifies bacteria previously cultured from a single culture result from each subject's medical record from subjects in the diabetic foot ulcer pool (D sample). Bacteria that were cultured and were not found using PRAPS include *Citrobacter *and *Pseudomonas*. % represents the percentage of the total sequences analyzed within the sample.

**Table 4:** Results obtained for the pressure ulcer sample (P) using pyrosequencing (PRAPS).

**Genus**	**Seq**	**%**	**Gram**	**Aerotolerance**	**Shape**
*Peptoniphilus *spp.	10543	38.38	+	Anaerobe	Cocci
*Serratia *spp.	5234	19.05	-	facultative anaerobe	Rod
*Peptococcus niger.*	3042	11.07	+	anaerobe	Cocci
*Streptococcus *spp.	3016	10.98	+	facultative anaerobe	Cocci
*Finegoldia magna*	1743	6.34	+	anaerobe	Cocci
*Dialister *spp.	1374	5.00	-	anaerobe	rod
*Pectobacterium *spp.	528	1.92	-	facultative anaerobe	rod
*Enterobacter *spp.	392	1.43	-	facultative anaerobe	rod
*Proteus *spp.	308	1.12	-	facultative anaerobe	rod
*Veillonella *spp.	186	0.68	-	anaerobe	cocci
UWB	141	0.51	Unk	unk	unk
UWB	121	0.44	Unk	unk	unk
*Clostridium *spp.	93	0.34	+	anaerobe	rod
*Corynebacterium striatum*	73	0.27	+	aerobe	rod
*Delftia *spp.	65	0.24	-	aerobe	rod
UWB	63	0.23	Unk	unk	unk
*Enterococcus *spp.	62	0.23	+	facultative anaerobe	cocci
*Staphylococcus *spp.	56	0.20	+	facultative anaerobe	cocci
*Hydrogenophaga *spp.	54	0.20	-	aerobe	rod
*Eggerthella lenta*	33	0.12	+	anaerobe	rod
UWB	31	0.11	Unk	unk	unk
UWB	31	0.11	Unk	unk	unk
*Prevotella *spp.	31	0.11	-	anaerobe	rod
*Varibaculum cambriense*	28	0.10	+	anaerobe	rod
*Actinomyces europaeus*	28	0.10	+	facultative anaerobe	rod
*Ferrimonas *spp.	27	0.10	-	facultative anaerobe	rod
*Bacillus *spp.	24	0.09	+	aerobe	rod
UWB	22	0.08	Unk	unk	unk
*Fusobacterium *spp.	22	0.08	-	anaerobe	rod
*Alcaligenes faecalis*	20	0.07	-	aerobe	rod
UWB	17	0.06	Unk	unk	unk
*Riemerella *spp.	15	0.05	-	aerobe	rod
UWB	14	0.05	Unk	unk	unk
*Stenotrophomonas *spp.	14	0.05	-	aerobe	rod
*Shewanella *spp.	11	0.04	-	facultative anaerobe	rod
*Eubacterium *spp.	10	0.04	+	anaerobe	rod

This table provides the identified genus of the bacteria found using pyrosequencing (PRAPS) in the P sample, the number of sequences corresponding to this genus, the known gram staining properties, the aerotolerance (anaerobic, anaerobic, or facultative anaerobic) nature of the genus, and the shape of the genus. UWB refers to unknown wound bacteria or sequences without significant similarity to database sequences. * The asterisk identifies bacteria previously cultured from a single culture result from each subject's medical record from subjects in the P pool. Bacteria that were cultured and were not found using PRAPS include *Acinetobacter, Leclercia, Morganella*, and *Pseudomonas*. The % represents the percentage of the total sequences analyzed within the sample.

### FRACS

The use of Full Ribosomal Amplification, Cloning and Sanger Sequencing (FRACS) has been utilized for at least 18 years to evaluate the biodiversity of environmental samples [70-72]. This method has also been used to evaluate the microbial diversity of environmental biofilms [73] and at least one study utilized this approach to evaluate the microbial diversity of diabetic foot ulcers [74].

When using FRACS for 200 random clones from each library and following vector screening and quality scoring, we found that between 179 and 194 sequences from each library could be analyzed. The break down of genotypes demonstrated similar trends (Table 5) as were observed using PRAPS. Strict anaerobes, cocci, and gram positives were scarce in the V group (Table 6). Facultative and strict anaerobic gram positive cocci were most prevalent in the D group (Table 7), and strictly anaerobic gram positive dominated the P group (Table 8). In V samples the predominant bacterial genus, identified by FRACS, were overwhelmingly *Pseudomonas *spp. (including *P. aeruginosa *and *P. fluorescens*) followed by *Enterobacter *spp. (including *E. cloacae*), *Stenotrophomonas maltophilia, Proteus *spp., and *Staphyloccoccus aureus *(Table 6). In the D sample the predominant species was overwhelmingly *Staphylococcus aureus *followed by *Anaerococcus lactolyticus, Anaerococcus vaginalis, Bacterioides fragilis, Finegoldia magna, and Morganella morganii *(Table 7). Finally, predominant bacteria in the P sample were *Peptoniphilus ivorii, Anerococcus *spp., *Streptococcus dysgalactiae*, and *Peptoniphilus *spp. (Table 8).

**Table 5:** Overview of the phenotypes of microbial populations as determined using shotgun Sanger sequencing (FRACS).

**Category**	**Pressure Ulcer seq**	**Pressure Ulcer sp**.	**Diabetic Foot Ulcer seq**.	**Diabetic Foot Ulcer sp**.	**Venous Leg Ulcer seq**	**Venous Leg Ulcer sp**.
aerobes	20	5	1	1	114	7
facultative anaerobes	21	8	102	15	69	15
anaerobes	143	13	75	15	7	3
Cocci	136	11	137	14	14	6
Rods	48	15	41	17	176	19
gram positive	153	14	136	15	21	9
gram negative	31	12	42	17	169	16
UWB	2	2	0	0	4	4

The data from Sanger sequencing (FRACS) is broken down into the number of sequences (seq) from each sample (P, D, and V) that were related to a given characteristic (anaerobe, gram positive etc). Also shown are the number of genera that were associated with each of the phenotypic categories. UWB refers to unknown wound bacteria or sequences without significant similarity to database sequences.

**Table 6:** Results obtained for venous leg ulcer sample (V) using shotgun Sanger sequencing (FRACS).

**Genus species**	**Number seq**	**%**	**Gram**	**Aerotolerance**	**Shape**
Pseudomonas sp*.	45	23.20	-	aerobic	rod
Pseudomonas aeruginosa.*	30	15.46	-	aerobic	rod
Pseudomonas fluorescens*	20	10.31	-	aerobic	rod
*Enterobacter *spp.	19	9.79	-	facultative	rod
*Stenotrophomonas maltophilia.*	14	7.22	-	aerobic	rod
*Enterobacter cloacae*	10	5.15	-	facultative	rod
Staphylococcus aureus*	7	3.61	+	Facultative anaerobe	cocci
*Proteus vulgaris*	6	3.09	-	Facultative anaerobe	rod
*Shewanella algae*	6	3.09	-	Facultative anaerobe	rod
*Proteus mirabilis.*	6	3.09	-	Facultative anaerobe	rod
UWB	4	2.06	unk	unk	unk
*Clostridium perfringens*	4	2.06	+	anaerobic	rod
*Klebsiella *spp.*	4	2.06	-	facultative	rod
Serratia marcescens *	3	1.55	-	aerobic	rod
*Clostridium *spp.	2	1.03	+	anaerobic	rod
*Helcococcus kunzii*	2	1.03	+	facultative anaerobe	cocci
*Staphylococcus *spp.*	2	1.03	+	Facultative anaerobe	cocci
*Enterobacter aerogenes*	2	1.03	-	Facultative anaerobe	rod
*Bacillus fusiformis.*	1	0.52	+	aerobic	rod
Enterococcus avium*	1	0.52	+	Facultative anaerobe	cocci
Enterococcus faecalis*	1	0.52	+	Facultative anaerobe	cocci
*Peptococcus *spp.	1	0.52	+	anaerobic	cocci
*Achromobacter xylosoxidans*	1	0.52	-	aerobic	rod
*Salmonella *spp.	1	0.52	-	Facultative anaerobe	rod
*Escherichia coli*	1	0.52	-	Facultative anaerobe	rod
*Shigella *spp.	1	0.52	-	Facultative anaerobe	rod

This table provides the identified genera of the bacteria found using shotgun Sanger sequencing (FRACS) in the V sample, the number of sequences corresponding to this genus, the known gram staining properties, the aerotolerance (anaerobic, anaerobic, or facultative anaerobic) nature of the genus, and the shape of the genus. UWB refers to unknown wound bacteria or sequences without significant similarity to database sequences. The asterisk * identifies bacteria previously cultured from a single culture result from each subject's medical record from subjects in the V pool. Bacteria that were cultured and were not found using FRACSS include *Acinetobacter, Citrobacter*, and *Corynebacterium*. The % represents the percentage of the total sequences analyzed within the sample.

**Table 7:** Results obtained for diabetic foot ulcer sample (D) using shotgun Sanger sequencing (FRACS).

**Genus species**	**Seq**	**%**	**Gram**	**Aerotolerance**	**Shape**
Staphylococcus aureus *	70	39.33	+	facultative	Cocci
*Anaerococcus lactolyticus*	16	8.99	+	anaerobic	Cocci
*Anaerococcus vaginalis*	15	8.43	+	anaerobic	cocci
*Bacterioides fragilis*	7	3.93	-	anaerobic	rod
*Finegoldia magna*	6	3.37	+	anaerobic	cocci
*Morganella morganii*	5	2.81	-	Facultative anaerobe	rod
Enterococcus faecalis*	5	2.81	+	facultative	cocci
*Peptoniphilus harei*	5	2.81	+	anaerobic	cocci
*Clostridium novyi*	4	2.25	+	anaerobic	rod
*Veillonella atypica*	4	2.25	-	anaerobic	cocci
*Abiotrophia para-adiacens*	4	2.25	+	anaerobic	cocci
*Veillonella parvula*	4	2.25	-	anaerobic	cocci
Citrobacter murliniae *	3	1.69	-	facultative anaerobe	rod
*Haemophilus *spp.	3	1.69	-	facultative	rod
*Clostridium *spp.	3	1.69	+	anaerobic	rod
*Haemophilus segnis*	3	1.69	-	faculative	rod
Enterococcus avium*	3	1.69	+	facultative	cocci
*Proteus *spp.*	2	1.12	-	Facultative anaerobe	rod
*Enterobacter aerogenes*	2	1.12	-	facultative	rod
*Dialister *spp.	2	1.12	-	anaerobic	rod
*Peptoniphilus asaccharolyticus*	2	1.12	+	anaerobic	cocci
*Enterobacter cloacae*	1	0.56	-	Facultative anaerobe	rod
Klebsiella oxytoca*	1	0.56	-	Facultative anaerobe	rod
*Escherichia coli*	1	0.56	-	facultative	rod
*Pseudoalteromonas *spp.	1	0.56	-	facultative	rod
*Porphyromonas levii*	1	0.56	-	anaerobic	rod
*Delftia acidovorans*	1	0.56	-	aerobic	rod
*Dialister invisus*	1	0.56	-	anaerobic	rod
Staphylococcus epidermidis*	1	0.56	+	facultative	cocci
*Staphylococcus *spp.*	1	0.56	+	facultative	cocci
*Granulicatella *spp.	1	0.56	+	anaerobic	cocci

This table provides the identified genera of the bacteria found using shotgun Sanger sequencing (FRACS) in the D sample, the number of sequences corresponding to this genus, the known gram staining properties, the aerotolerance (anaerobic, anaerobic, or facultative anaerobic) nature of the genus, and the shape of the genus. The asterisk * identifies bacteria previously cultured from a single culture result from each subject's medical record from subjects in the D pool. Bacteria that were cultured and were not found using FRACSS include *Pseudomonas, Serratia*, and *Streptococcus*. The % represents the percentage of the total sequences analyzed within the sample.

**Table 8:** Results obtained for the pressure ulcer sample (P) using shotgun Sanger sequencing (FRACS).

**Genus species**	**Seq**	**%**	**Gram**	**Aerotolerance**	**Shape**
*Peptoniphilus ivorii*	51	27.42	+	anaerobic	cocci
*Anaerococcus lactolyticus*	20	10.75	+	anaerobic	cocci
*Anaerococcus vaginalis*	16	8.60	+	anaerobic	cocci
Streptococcus dysgalactiae*	14	7.53	+	facultative	cocci
*Peptoniphilus harei*	14	7.53	+	anaerobic	cocci
*Peptococcus niger*	12	6.45	+	anaerobic	rod
*Serratia marcescens*	12	6.45	-	aerobic	rod
*Finegoldia magna*	10	5.38	+	anaerobic	cocci
*Peptoniphilus indolicus*	7	3.76	+	anaerobic	cocci
*Clostridium hathewayi*	4	2.15	+	anaerobic	rod
*Prevotella buccalis*	3	1.61	-	anaerobic	rod
Pseudomonas aeruginosa*	3	1.61	-	aerobic	rod
UWB	2	1.08	Unk	unk	unk
*Dialister invisus*	2	1.08	-	anaerobic	rod
*Dialister *spp.	2	1.08	-	anaerobic	rod
*Delftia acidovorans*	2	1.08	-	aerobic	rod
Pseudomonas fluorescens*	2	1.08	-	aerobic	rod
*Helcococcus kunzii*	1	0.54	+	facultative anaerobe	cocci
Enterococcus faecalis*	1	0.54	+	facultative	cocci
Staphylococcus epidermidis*	1	0.54	+	facultative	cocci
Streptococcus pyogenes*	1	0.54	+	facultative	cocci
*Clostridium perfringens*	1	0.54	+	anaerobic	rod
*Klebsiella granulomatis*	1	0.54	-	Facultative anaerobe	rod
*Klebsiella pneumoniae*	1	0.54	-	Facultative anaerobe	rod
Proteus vulgaris*	1	0.54	-	Facultative anaerobe	rod
*Porphyromonas uenonis*	1	0.54	-	anaerobic	rod
*Pseudomonas *spp.*	1	0.54	-	aerobic	rod

This table provides the identified genera of the bacteria found using shotgun Sanger sequencing (FRACS) in the P sample, the number of sequences corresponding to the given genus, the known gram staining properties, the aerotolerance (anaerobic, anaerobic, or facultative anaerobic) nature of the genus, and the shape of the genus. UWB refers to unknown wound bacteria or sequences without significant similarity to database sequences. The asterisk * identifies bacteria previously cultured from a single culture result from each subject's medical record from subjects in the P pool. Bacteria that were cultured and were not found using FRACSS include *Acinetobacter, Enterobacter, Leclercia *and *Morganella*. The % represents the percentage of the total sequences analyzed within the sample.

### PRADS

The use of a Partial Ribosomal Amplification, DGGE, and Sanger sequencing (PRADS) has been utilized extensively to study microbial diversity [75-80]. This approach has also been used extensively to study the microbial diversity of biofilms [81-84] and has even been used to study clinical biofilms [85]. The primary use of DGGE by itself is to provide an indication of diversity. The number of bands seen in a gel, in many cases, can provide a relative measure of the number of different bacteria present. It is understood that each of the bands seen in a DGGE gel can represent multiple species and even the same species can be represented by multiple bands as we observed in the current study (data not shown).

By excising the predominant bands from each sample, cloning them into a vector, and sequencing them, the identity of the bacteria from each band are identified. In the V sample the primary bacteria were *Enterobacter, Pseudomonas*, and *Proteus *spp. (Table 9). This is similar to the results seen using both FRACSS and PRAPS. In the D sample, PRADS identified *Pseudomonas, Haemophilus, Citrobacter*, and *Stenotrophomonas *as the predominant species (Table 9). In the P sample the primary species identified was *Serratia, Dialister*, and *Peptococcus *spp. (Table 9).

**Table 9:** Results obtained for each of the samples using DGGE band extraction and sequencing (PRADS).

**V seq**	**V genus**	**D seq**	**D genus**	**P seq**	**P genus**
18	*Enterobacter *spp.*	13	*Pseudomonas *spp.*	34	*Serratia *spp.
17	*Pseudomonas *spp.*	12	*Haemophilus *spp.	13	*Dialister *spp.
4	*Proteus *spp.	11	*Citrobacter *spp.*	10	*Peptococcus *spp.
2	*Klebsiella *spp.*	11	*Stenotrophomonas *spp.	3	*Pseudomonas *spp.*
2	*Pectobacterium *spp.	10	*Morganella *spp.	2	*Citrobacter *spp.
2	*Erwinia *spp.	10	*Staphylococcus *spp.	2	*Morganella *spp.
1	*Serratia *spp.*	5	*Acinetobacter *spp.	2	*Proteus *spp.*
1	UWB	5	*Acinetobacter *spp.	1	*Haemophilus *spp.
1	*Haemophilus *spp.	4	*Morganella *spp.	1	*Klebsiella *spp.
		4	*Proteus *spp.*	1	*Leminorella *spp.
		3	*Delftia *spp.	1	*Pectobacterium *spp.
		3	*Obesumbacterium *spp.	1	*Peptoniphilus *spp.
		2	*Dialister *spp.	1	*Prevotella *spp.
		2	*Mannheimia *spp.	1	UWB
		1	*Comamonas *spp.		
		1	*Grimontia *spp.		
		1	*Klebsiella *spp.*		
		1	*Macrococcus *spp.		
		1	*Methylophaga *spp.		
		1	*Pantoea *spp.		
		1	*Pectobacterium *spp.		
		1	*Rahnella *spp.		
		1	*Serratia *spp.*		
		1	*Streptococcus *spp.*		
		1	UWB		

This table provides the identified genus of the bacteria found in each of the samples as determined by DGGE band excision, cloning and sequencing (PRADS). The number of sequences corresponding to this genus is provided. The physiological aspects of these isolates are described elsewhere in most cases. UWB refers to unknown wound bacteria or sequences without significant similarity to database sequences.

#### Culturing

A review of the literature identifies *Staphylococcus *spp. as the predominant organisms associated with wounds based upon culturing [86-88]. As discussed in the introduction, this is primarily due to the ability of this bacterium to be propagated in culture media under typical laboratory conditions. *Pseudomonas *spp., another easy to culture bacteria is also frequently isolated from chronic wounds using culture methods [89,90]. Other species that have been most consistently identified in association with chronic wounds include *E. coli, Enterobacter cloacae, Klebsiella, Streptococcus, Enterococcus. *and *Proteus *spp. [25,58,86,88,90-95]. One notable commonality of the above organisms is the ease with which they can be cultured in standard laboratory growth media under aerobic conditions. These claims are upheld by the data presented here, as all of the isolates identified using the clinical cultures are relatively easy to propogate under aerobic conditions in standard laboratory media (Table 10).

**Table 10:** Bacteria cultured during standard of care from three wound groups.

**Subjects **	**V group**	**Subjects**	**D group**	**Subjects**	**P group**
4	*Enterococcus*	2	*Citrobacter*	4	*Staphylococcus*
3	*Staphylococcus*	2	*Enterococcus*	2	*Streptococcus*
2	*Enterobacter*	2	*Klebsiella*	2	*Enterococcus*
2	*Pseudomonas*	2	*Serratia*	1	*Escherichia*
1	*Klebsiella*	2	Staphylococcus	1	*Leclercia*
1	*Serratia*	2	*Streptococcus*	1	*Proteus*
1	*Citrobacter*	1	*Proteus*	1	*Pseudomonas*
1	*Acinetobacter*	1	*Pseudomonas*	1	*Acinetobacter*
		1	*Escherichia*	1	*Enterobacter*

Subject's medical records were examined, and the positive culture nearest to the date of sample collection for molecular analysis was considered. All thirty (30) subjects except for one (1) subject in the P group had a culture recorded in their medical records. The number of subjects within each group to culture positive for a bacterial genus is listed next to the genus. Many of the subjects were cultured for both aerobes and anaerobes, but the clinical laboratories reported no obligate anaerobes.

Culture results for 29 of the 30 subjects were found by auditing the subjects' past and current longitudinal medical records. The bacteria isolated are indicated for comparative purposes in Tables 2, 3, 4 and 6, 7, 8 and reported fully in Table 10. Cultures were positive for some bacteria that were not identified using PRAPS, FRACS, or PRADS. In the V group, *Citrobacter *was identified via culture and was not discovered with molecular methods. In the D group, all bacteria that were cultured were identified using molecular methods. In the P group, *Acinetobacter *and *Escherichia *spp. were cultured but not identified via molecular methods. As noted in the methods, the samples used for culturing were not necessarily collected in parallel with the samples collected for molecular analyses. Continuous longitudinal attempts to culture anaerobic bacteria had also been made and were unsuccessful for any of these patients. This highlights the problems with laboratory culture methods especially with the P type chronic wound, which is predicted to be primarily anaerobic using each of the molecular methods.

### Comparison to normal flora

The microbial flora of normal skin is also considered complex. A bacterial diversity study of normal skin flora from 6 healthy subjects was performed using molecular methods [96]. It was found that there were hundreds of bacterial species among the individuals. The conclusions of this study indicated that normal flora of skin is highly diverse and only a few bacteria are common among the individuals. These included *Propionibacteria*, *Corynebacteria*, *Staphylococcus*, and *Streptococcus *spp. [96]. These results were largely corroborated by a previous study which collected and analyzed swabs from the forehead of 5 individuals. This study also found *Staphylococcus *and *Propionibacteria *spp. as well as high prevalence of *methylophilus *spp. [97]. Few other studies were found evaluating normal skin microflora suggesting that more extensive studies of healthy skin microflora are also needed. The primary bacteria that occur on healthy skin also correlate to the primary bacteria cultured from wound biofilms. Future studies are needed to evaluate the normal flora and chronic wound biofilm flora from the same patients.

### Anaerobes in pathogenic biofilms

In relation to anaerobes, the literature is now beginning to show their importance in chronic wound pathogenic biofilms. Even though such wounds are typically exposed to air [88] anaerobes may be most prevelant physiological type for a given wound or a given wound type as shown in this study. Many of these newer studies show the importance of anaerobes such as *Peptostreptococcus, Prevotella, Finegoldia *and *Peptoniphilus *spp. [88,89,91,93,94,98], which were also seen as important in the current survey. However, as noted previously, only a few studies have looked at the populations of bacteria in various wound types. Bowler et al [88] evaluated venous leg ulcers using cultural isolation techniques that included special considerations for the propogation of anaerobes. They found that anaerobes represented 49% of the total microbial composition in such wounds. This does not agree with our analyses of the V type ulcer, which showed only 1.6% of sequences were matched to anaerobes. However, almost 30% of the sequences from D and 62% of sequences from P wound types were matched to anaerobes. An interesting observation is that the differences in the functional diversity of the pathogenic biofilms may suggest important differences in the physiology of these three types of wounds. As indicated previously, the pathophysiology of a wound type may select for certain physiological or functional populations within the associated pathogenic biofilm. Thus, the bacterial populations which are prevelant might in turn suggest differences in management of the individual CWPB are necessary.

As has been demonstrated in the laboratory [59,99], anaerobes may cope with the toxic effects of oxygen by interacting with aerobic or facultative anaerobic bacterial populations in a symbiotic manner as part of a process known as coaggregation. Aerobic species may consume oxygen and create localized environments, allowing the obligate anaerobes to gain advantage when in close proximity. The Lewandowski lab has also shown that oxygen only penetrates microns into the surface of biofilms suggesting that internal regions may support only anaerobes and facultative anaerobes [100]. Such synergistic interactions and advantages of biofilm phenotype have been shown for specific aerobes and anaerobes. Using animal models it has also been shown that mixtures of anaerobic and aerobic bacteria have been shown to produce disease states which cannot be reproduced by the individual species alone [61,101,105]. These findings suggest a complexity to the host-pathogen interaction that adds a new dimension to Koch's postulates. These finding also dramatically highlight the failings of culture methods to identify major populations of importance within each of the wound types.

## Conclusion

The primary contrasts we see in these data are the differences found in bacterial populations within wound types using culture (Table 10) and molecular analyses (Tables 1, 2, 3, 4, 5, 6, 7, 8, 9). Here we show that culturing failed to identify major contributing populations, especially strict anaerobes, within the given wound types. Standard culturing techniques are inherently biased as they only examine only the 1% of all microorganisms which are able to grow fairly rapidly in pure culture. Culturing also requires several days before the culturable bacteria can be identified whereas molecular methods such as PCR can typically be completed within several hours. In addition, certain of the isolates we have shown are primary populations within a wound type, may never be cultured in the laboratory due to reduced metabolic activity, obligate cooperation with other bacteria, requirements for specialized nutrients, or growth in specific environmental conditions [106]. Molecular methods unlike culture methods also have more potential to provide quantitative data. Arguably, we have shown that molecular methods will allow populations residing within biofilms to be more fully characterized. The continued development of molecular methods may lead to vastly improved tools for diagnostics that will identify and provide quantification of the diverse species potentially present in chronic wounds thereby allowing physicians to better tailor their treatment to each patient's unique pathogenic biofilm populations. This dramatically highlights the need to move microbiological analyses of chronic wound pathogenic biofilms toward molecular approaches.

If clinicians can gain a better understanding of the wound's microbiota, it will give us a greater understanding of the wound's ecology and will allow us to better manage the wound. It is important to consider the bacterial populations within pathogenic biofilms for many reasons. These reasons typically relate to the fact that the higher bacterial population diversity within a pathogenic biofilm provides the bacterial community as a whole with an enhanced ability to persist and thrive in a variety of antagonistic situations, even in spite of combined host and medicinal attack [107]. The current study has shown that a wide variety of bacteria with different physiological and phenotypic preferences are common as part of pathogenic biofilm communities in chronic wounds. Additionally, we can see that different types of wounds may have different bacterial populations that are prevalent. Thus, the CWPB in one wound or wound type may indicate a therapy that is different than that indicated in another wound or wound type. As noted previously, such conclusions are beyond the scope of this survey but are suggested by the results. These conclusions also provide direction for future research. The use of traditional culture techniques are widely used, but we and others have consistantly demonstrate that they are not likely to be the best way to elucidate the bacterial populations within CWPB.

## Methods

Biofilms from a total of 30 chronic wound patients were included in this survey study. A total of 30 chronic wounds were sampled and grouped into one of three categories of wounds: venous leg ulcers (V), diabetic foot ulcers (D), or pressure ulcers (P). To identify the microbial populations that occur in these types of wounds and to gain a preliminary understanding of their relative importance, we took advantage of three powerful molecular approaches that included PRADS [108], FRACS [109], and PRADS [110]. These techniques allowed the bacterial diversity that occurs within these CWPB types to be evaluated.

### Protocol for subject enrollment, DNA extraction, and Sample Preparation

Under the guidance of IBR protocols, chronic wounds of 30 subjects treated at the Southwest Regional Wound Care Center (Lubbock, Texas) were debrided as per standard of care; the debridement samples were collected with sterile tools into sterile collection tubes and frozen until processing for DNA extraction. Samples from 10 subjects with venous leg ulcers, 10 subjects with diabetic foot ulcers, and 10 subjects with pressure (decubitus) ulcers were included in this study. Debridement samples (300 mg ± 150 mg) were collected into Lysing Matrix E tubes from the FastDNA^® ^SPIN for Soil Kit from MP Biomedicals LLC (Solon, OH). The tubes were frozen at -70°C until DNA extraction could be performed. When DNA extraction was performed, the samples were removed from the freezer and allowed to thaw at room temperature. Subsequently, the DNA extraction protocol for the kit was followed with the exception that human debridement samples were used in place of soil samples. The extracted sample DNA was stored at -70°C. After measuring the relative concentration of bacterial DNA present based upon 16s quantitative PCR using 16S Universal Eubacterial primers 530F (5'-GTG CCA GCM GCN GCG G) and 1100R (5'-GGG TTN CGN TCG TTG), the 10 samples from each of the three wound groups were pooled at equal bacterial DNA ratios to create three pools of DNA, each representing a major category of chronic wound.

### Partial ribosomal amplification and pyrosequencing (PRAPS)

The modified 16S Eubacterial primers 530F and 1100R were used for amplifying the 600 bp region of 16S rRNA genes. The primer pair used for 454 Amplicon Sequencing was designed with special Fusion Primers at the 5' end of each primer as follows: 530F-A (5'-GCC TCC CTC GCG CCA TCA GGT GCC AGC MGC NGC GG) and 1100R-B (5'-GCC TTG CCA GCC CGC TCA GGG GTT NCG NTC GTT G). All wound DNA samples were diluted to 100 ng/μl. A 100 ng aliquot of sample DNA was used for a 50 μl PCR reaction. HotStarTaq Plus Master Mix Kit (QIAGEN, CA, USA) was used for PCR under the following conditions: 94°C for 3 minutes followed by 32 cycles of 94°C for 30 seconds; 60°C for 40 seconds and 72°C for 1 minute; and a final elongation step at 72°C for 5 minutes. PCR products were purified using the PSI Ψ Clone PCR 96 Kit (Princeton Separations Inc, Freehold, NJ). Following PCR amplification, samples were normalized in concentration and sent on dry ice to the Medical Biofilm Research Institute [111] for pyrosequencing using genome sequencer FLX system's standard amplicon sequencing protocols (F. Hoffmann-La Roche Ltd, Basel, Switzerland).

### Full ribosomal amplification, cloning and Sanger sequencing (FRACS)

The Eubacterial 16S primers 27F (5'-AGA GTT TGA TCM TGG CTC AG) and 1525R (5'-AAG GAG GTG WTC CAR CC) were synthesized by Integrated DNA Technologies (Coralville, IA). These primers amplify roughly 1500 bp spanning almost the entire 16S gene. A total of 100 ng of sample DNA was used for each 50 μl PCR reaction. HotStarTaq Plus Master Mix Kit (Qiagen, Valencia, CA) was used for PCR using the following conditions: 94°C for 3 minutes followed by 35 cycles of 94°C for 30 seconds, 52°C for 40 seconds, and 72°C for 2 minutes. A final elongation step at 72°C for 10 minutes was also included. The amplified fragments were subcloned into the pGEM-T Easy Vector (Promega, Madison, WI) and transformed into a competent *E. coli *K12 strain. Following blue/white screening using standard methods, 200 clones from each library were isolated and subcultured. The plasmid DNA was extracted by using a QIAprep Spin Miniprep Kit (Qiagen, Valencia, CA). Bidirectional sequencing using T7 and SP6 primers was performed by Agencourt Technologies (Beverly, MA).

### Partial Ribosomal Amplification, DGGE, and Sanger sequencing (PRADS)

The 16S Eubacterial primers 1070F (5'-ATG GCT GTC GTC AGC T) and 1492R+GC (5'-GCC GCC TGC AGC CCG CGC CCC CCG TGC CCC CGC CCC GCC GCC GGC CCG GGC GCC TTA CCC TTG TTA CGA CTT) were synthesized by Integrated DNA Technologies (Coralville, IA). These primers produced an approximately 450 bp 16S ribosomal DNA (rDNA) amplicon with a GC clamp to be analyzed by denaturing gradient gel electrophoresis (DGGE). PCR reactions (50 μl of sample DNA) were performed using 2× PCR Master Mix (Promega, Madison, WI). Each reaction mixture consisted of 1.5 mM MgCl2, 200 μM of each dNTP, 0.5 μM of both the 1070F and 1492R+GC primers, 0.025 U/μl Taq DNA polymerase, and 100 ng template DNA.

Denaturing gradient gel electrophoresis (DGGE) was performed on the 16S amplicons described above using the DCode™ DGGE system (Bio-rad). A 40%–70% denaturing gradient was optimal for separation of the approximately 450 bp 16S amplicons, where 7 M urea and 40% formamide is defined as 100%. Gels also contained an 8%–12% acrylamide gradient with a 12% native stacking gel. Different volumes of each sample were loaded for optimal visualization of bands with varying intensities. The gel was run at 60 V for 20 hours and was then stained with SYBR Gold^® ^(Molecular Probes, Invitrogen, Carlsbad, CA) and visualized with a FluorChem™ 8800 fluorescence imager (Alpha Innotech Inc. San Leandro, CA). Nineteen predominant bands that could be visualized by eye were excised using a sterile, disposable scalpel and placed in 20 μl TE buffer (Figure 1). This included 4 bands from the venous leg ulcer group (group V), 8 from the diabetic foot ulcer group (group D), and 6 from the pressure ulcer group (group P).

The TOPO TA Cloning^® ^kit (Invitrogen Inc. Carlsbad, CA) was used to clone the DNA from the excised DGGE bands (pCR^® ^2.1-TOPO^® ^vector and One Shot Chemically Competent *E. coli *cells). The maximum amount of DNA (4 μl diffused DNA in TE buffer) was used in each of the cloning reactions following the manufacture's instructions. Twelve clones per band were selected and grown overnight in 250 μl LB broth containing 50 μg/ml kanamycin in 96 well plates. The same 50 μl PCR reaction was prepared as described above using the M13F and M13R primer set instead of 1070F and 1492R+GC and 5 μl of the overnight culture was added to each 50 μl PCR reaction as the template DNA. The initial 96°C denaturing step was sufficient to rupture the *E. coli *cells, releasing its DNA as the starting template. These PCR products were then sequenced by the University of Washington's High-Throughput Genomics Unit (Seattle, WA).

### Culturing

Samples were not collected in parallel with samples collected for the PRAPS, FRACS, or PRADS; but culture results were instead collected retrospectively from the subjects' medical records. All samples were collected under IBR protocols. This information is provided as a contrast to the type of data normally collected from wounds. Results were examined from each subject's medical record. The culture results were collected usually within a week of the samples that was collected for the PRAPS, FRACS, and PRADS analyses. Sharp debridement from the subject's wound was placed in thioglycolate broth with indicator (Hardy Diagnostics, Santa Maria, CA) and incubated at 35°C for up to 24 hours with the lid tightly closed before being transferred to a CLIA certified microbiology laboratory for thorough aerobic and anaerobic culture analyses. Bacteria were identified using Gram stains, non-selective and selective/differential media, and biochemical tests.

### Bioinformatics

Assembly, including vector scanning, quality analysis, and consensus calling, of sequencing data was performed using Seq-Man Pro assembler (DNAstar Madison, WI). Sequences were assembled using SeqMan Pro Assembler at 96% similarity, match size of 25, match spacing of 50, minimum sequence length of 100, 0.0 and 0.7 gap and gap extension penalties, and a minimum mismatch of 8 at end bases. Multiple alignments were performed with MegAlign (DNAstar Madison, WI). BLAST analyses were performed using WND.BLAST [112] and a custom 16S ribosomal database derived from RDPII version 9 [113,114]. The database was first parsed with a custom script to provide genus and species names as hit definitions following BLAST analyses. For FRACS E-values < 10E-100 were considered acceptable for determining genus, while hits of E = 0.0 and associated full alignments > 1400 bp were considered acceptable for determining genus and species. For PRADS and PRAPS all species determinations are considered putative, E-values of 10–100 are considered acceptable for determining genus, while E-values better than 10–140, along with full input sequence alignments, and genus/species agreement of all similarly scoring top hits were required to list a putative species.

## Authors' contributions

SED was responsible for primary conception of the methods, project management, data interpretation, and development and approval of the final version of the manuscript. YS helped develop methods, performed most of the laboratory studies, and data compilation. PS and GJ performed DGGE and all associated writing and data interpretations. DR helped with interpretation of results, compiling data, and writing of early drafts of the manuscript. RW was vital in developing the project concepts, interpretation of results and approval of final draft of the manuscript.
